# Addressing convergence in the emerging era of structural phylogenetics

**DOI:** 10.3389/fmolb.2026.1833246

**Published:** 2026-05-01

**Authors:** Jamie D. Dixson, Rajeev K. Azad

**Affiliations:** Department of Biological Sciences and BioDiscovery Institute, University of North Texas, Denton, TX, United States

**Keywords:** AlphaFold, convergence, convergent evolution, foldtree, homoplasy, protein structure, pseudogene

## Introduction

### Background

Convergent evolution is widespread in biological systems, and any phenotypic trait may experience convergent evolution (Bower et al., 2021; McGhee, 2011; Sackton and Clark, 2019; Vowles and Amos, 2004). This is true for macromorphological traits such as wings in birds, bats and insects (McGhee, 2011) or meristic traits such as scale counts in fish (Bower et al., 2021), as well as for micromorphological traits such as the microstructures of the sensilla in distantly related insects (Keil, 1997) and the micro-ornamentation patterns of certain mollusks (Asato et al., 2022). It is also true in the ultrastructural realm at the transition point from morphological to molecular as evidenced by the fact that cyanobacteria, green algae, and land plants all independently evolved similar stacked thylakoid membranes (Mareš et al., 2019). In-fact the list of morphological traits that have evolved convergently is quite extensive regardless of whether those traits are macromorphological, micromorphological or ultrastructural (McGhee, 2011).

Considering the numerous examples of convergence in the higher structural realms, it stands to reason that convergence might also be prominent when considering molecular structures (Rokas and Carroll, 2008). While there are numerous examples where molecular convergence is plausible (Vowles and Amos, 2004), it is often either ignored or discounted in favor of remote homology (Moi et al., 2025; Nagano et al., 2002). That is understandable since convergence and remote homology can sometimes be indiscernible using sequence data (Dixson et al., 2025; Dixson and Azad, 2025). One example where remote homology is assumed but convergence is plausible is the TIM-barrel fold (Nagano et al., 2002; Romero-Romero et al., 2021). This fold is found in numerous isolated enzyme families and does not show any appreciable sequence similarity among those families. Convergence is a plausible scenario often in the realm of low sequence similarity, yet remote homology is frequently posited despite the lack of overwhelming evolutionary evidence to support it (Nagano et al., 2002). The plausibility of alternative scenarios including convergence is often not assessed. This begs the question, why is there a tendency to assume remote homology at the molecular level but not at the macromorphological, micromorphological or ultrastructural levels?

### Clarification of convergent evolution

**FIGURE 1:**
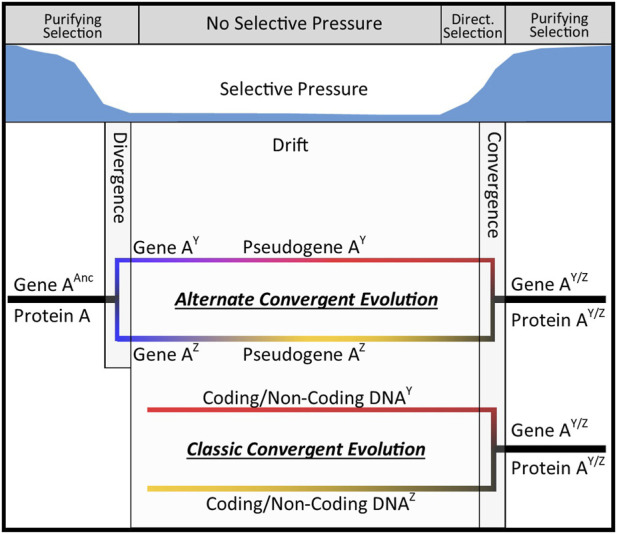
This diagram illustrates that lineage independence is not necessary for convergent evolution to occur. In classic convergent evolution, the precursor genetic material must occur on unrelated lineages. An alternate form of convergent evolution can occur if the precursor genetic material once encoded the same protein followed by relief of selective pressure for a period of divergence followed by regain of function when selective pressure is restored.

Before delving deeper into the discussion of convergent molecular evolution, it is important that a few clarifications be made. First, it is often stated that convergent evolution is the evolution of similar traits in independent or unrelated lineages (Castoe et al., 2010; 2009). This is inconsistent with the concept put forth by Darwin and widely accepted belief that all organisms on earth are related and thus evolutionarily dependent, (Darwin, 1859; Delaye and Becerra, 2012), though a contrarian view of multiple independent origins also exists (Raup and Valentine, 1983). Also, this is not just a linguistic distinction, it has far-reaching implications in convergent evolutionary analyses, and it is often overlooked. Additionally, the distinction between convergent and parallel evolution is a sticking point for some. For example, consider this conceptual example. Species X may undergo a speciation event resulting in two descendent species (Species Y and Z). That event may then be followed by a change in environmental conditions which relieves purifying selection for protein A in both descendent taxa. That then allows the gene encoding protein A to become a pseudogene in both taxa. Those pseudogenes, which are no longer subject to selective pressure, then diverge independently in species Y and Z. Later, and after environmental conditions revert to their original state, the two, now divergent, pseudogenes can become resurrected due to new selective pressure. The new protein A^Y^ and protein A^Z^ are products of descendants of independent and divergent pseudogenes and likely differ in both gene and protein sequences. Despite that, they may once again achieve the original function and thus converge upon that phenotype. Therefore, this is not a case of parallel evolution as it would have been if the pseudogene sequences had not diverged (Rokas and Carroll, 2008). This is also not merely purifying selection because the two lineages independently existed for a period as divergent pseudogenes. In this case, the two lineages are clearly related, yet convergence also clearly occurred. Therefore, a more appropriate definition for convergent evolution is the evolution of similar phenotypes in divergent lineages regardless of the degree of convergence. This definition, unlike the more common definitions, removes the requirement for lack of ancestry and fully embodies convergence existing in a state of flux. The scenario described above is illustrated in Figure 1 for clarity. Incidentally, that state of flux is key to the concept that pseudogenes can serve as a diversity bank considering ever-changing selective pressures (Balakirev and Ayala, 2003).

The other evolutionary scenario that should be in discussions concerning convergent evolution and structural phylogenetics is horizontal gene transfer (HGT). HGT, a physical process of gene transfer from one organism to another across a species boundary, is often invoked to explain aberrant phylogenetic placements (Azad and Lawrence, 2012). However, it should not be considered in exclusion to convergent evolution. On the contrary, it is just one of the many contributing factors to the overall mode of evolution. In other words, HGT may in some cases be the process by which a trait begins evolving or converging in a divergent taxon.

### Structural phylogenetics and convergent evolution

Through the years, there have been numerous attempts to use the structures of proteins to reconstruct phylogenies (Agarwal et al., 2009; Breitling et al., 2001; Bujnicki, 2000; Eventoff and Rossmann, 1975; Herman et al., 2014; Johnson et al., 1990; Khilji et al., 2025; Moi et al., 2025; Sun et al., 2021). Most of those attempts have been extremely limited in scope due to the minimal number of available experimental structures. This was a result of the time, cost and complexity of experimental protein structure determination. Recently, restrictions on structural phylogenetic reconstruction were mitigated with the development of highly accurate algorithms for the *de novo* determination of protein structures; most notably, the AlphaFold program (Jumper et al., 2021; Varadi et al., 2022). Following the production of millions of AlphaFold structures, there has been a rapid expansion in the interest in producing phylogenies from those structures. This, notably, spawned the development of FoldTree, a tool that can directly use AlphaFold structures to produce structural distance-based phylogenetic trees (Moi et al., 2025). While FoldTree was not the first tool to produce structure-based distances that could be used in the reconstruction of phylogenetic trees (Holm and Sander, 1993; Zhang and Skolnick, 2005), it was the first to be designed and validated for the reconstruction of trees from large numbers of AlphaFold structures. In the validation experiments, FoldTree was able to accurately produce known phylogenetic topologies from well-defined benchmark data. Those validation experiments clearly illuminate the utility of the method (Moi et al., 2025).

One potential issue with FoldTree, or any other reconstruction method that uses experimental or *de novo* structures, is that protein structures are often not considered within the realm of purely phenotypic traits. Like other phenotypic traits, protein structures are also subject to unexpected similarities in diverse lineages because of convergent evolution. A full discussion of the potential problems that convergent evolution could cause in phylogenetic analyses using protein structures were not addressed in the FoldTree validation paper. Instead, there was only mention of convergent evolution as a less plausible contributing factor to the observed tree topology in the sequence-based tree for the RRNPPA receptors (Moi et al., 2025). Despite this, it is prudent that we, as a scientific community, explore the effects of convergence on the topologies produced using any method focused on the reconstruction of phylogenetic trees using protein structure data. This is especially true for structural distance-based techniques, such as FoldTree, that use continuous protein structures and perform tree reconstruction based on pairwise distances between structures. FoldTree compresses the evolutionary signal into a single metric based on structural encodings that could, in some instances, encode convergent structural features, similar to encoding of evolutionarily related features, thus confounding inferences. One might argue that convergent evolution could be revealed by parsimoniously mapping characters onto a FoldTree tree (small parsimony). However, the indiscrete character delineation and inability to discriminatively encode structurally similar but evolutionarily distinct features limit the application of small parsimony to convergence detection. This serves to illuminate a long-recognized vulnerability of structural tree reconstruction methods when considering the characterization of convergence of phenotypic traits (Klingenberg and Gidaszewski, 2010). It also highlights that an alternative method to identify convergence in protein structure trees is necessary.

As previously asserted, the degree of convergence is an important factor that is in a constant state of flux. The example above demonstrates that convergence need not be extensive to exist. However, the aforementioned example also creates a paradox because if the extant Species Y and Species Z are sampled, the structural phylogenetic tree would likely place them in a very similar location to where they would have been had they not gone from active gene product to pseudogene back to active gene product and also have experienced convergent evolution. In other words, a phylogenetic tree may not reflect the dynamics of, and accompanying changes in, evolution. Since convergence can have effects across a wide spectrum of intensity, it is imperative that any method that addresses the effects of convergence on phylogenetic topologies also be able to address that full spectrum. There must be a certain level of pre- or post-processing of structural phylogenetic trees or the underlying data that either corrects for convergence or at minimum illuminates areas in the trees where convergence might be suspected. This is especially true for large phylogenetic trees, with scores of taxa, making it nearly impossible for manual examination of all the placements. In such cases, an algorithmic method to highlight potentially convergent scenarios would be valuable.

Previously, we demonstrated that a physicochemical technique, Molecular Weight Hydrophobicity Physicochemical Dynamic Time Warping, (MWHP PCDTW) could be used to illuminate randomly produced, highly divergent proteins that were well within the root mean deviation (RMSD) limits for remote homologs (Dixson and Azad, 2025). In other words, they mimicked what might be expected from either extreme remote homology or extreme convergence. Since there is no model to produce convergent protein datasets, this random model was used as a stand-in for convergent evolution. We then deployed the technique, which requires only the amino acid sequences, to identify parts of a FoldTree-produced cytochrome P450 phylogenetic tree where convergence should be considered. Those areas of enhanced indication of convergence were in some cases also supported by literature-based functional data demonstrating the utility of MWHP PCDTW in finding convergence in a phylogenetic tree (Dixson et al., 2025). In the validation study underlying that assumption, the technique showed clear separation between actual remote homologs and synthetically produced protein sequences that showed RMSD similarity to an extent that they could be (mis)construed as homologs at the structural level (Dixson and Azad, 2025). These data were then used to train an SVM classifier that was later used to classify extant taxa as either likely to have been derived from a convergent or random process or not (Dixson et al., 2025). That classification was presented as a probability rather than a boolean class, thus functioning as a graded indicator of convergence rather than a basic classifier. This is key in the large-scale analysis of scores of potentially remote homologs where manual curation becomes increasingly difficult due to the dataset size. This demonstrates the usefulness of such classifiers in identifying convergence in large trees created using protein structure-based tree reconstruction methods such as FoldTree, obviating the need for delineating purely discrete character boundaries to detect convergence, which presents a different set of challenges and has its own inherent limitations. This makes MWHP PCDTW a logical and practical choice for the identification of potential convergence in protein structure trees, at a much large scale.

## Conclusion

Assumptions are necessary in all evolutionary analyses. The most common and high confidence assumptions are based on our current understanding of the evolutionary process. However, the tendency to discount or even ignore some processes, positing that they make such small contributions to the overall process that the implications are negligible, is not uncommon. This appears to underly the general consideration of convergence in structural phylogenetic analyses.

There have been several studies conducted recently concerning structural phylogenetics. In many of those studies the authors either only briefly mention convergent evolution (Khilji et al., 2025) or consider it to play a relatively modest role (Kim et al., 2025; McShea et al., 2025; Moi et al., 2025). However, other authors not only mention convergent evolution but also emphasize the difficulties it presents in interpreting phylogenetic trees created from protein structures (Dixson et al., 2025; Puente-Lelievre et al., 2025). These very recent contributions to the field of structural phylogenetics demonstrate the timeliness of addressing convergent evolution in structural phylogenetics now rather than retroactively addressing it later.

We must consider that convergence can exist across a wide spectrum. Ignoring the relative contribution of convergent evolution to the overall extant phylogenies of proteins diminishes the very spirit of efforts currently being made towards robust and reliable phylogenies. It would, therefore, be prudent to revisit and reexamine convergent evolution and incorporate the newly gained or refined understanding from the large-scale omics data analyses in the field, lest we risk repeating the mistakes of the past when phenotypic traits were erroneously attributed to divergent evolution but later emphasized to likely be the result of convergence (Bower et al., 2021; McGhee, 2011; Vidal and Hedges, 2005; Wootton, 1992).

Given the ambiguity about the overall impact of convergent evolution on evolutionary processes, it would be wise to reflect on the insights of Alexey Murzin, from nearly 30 years ago, quoting, “I think that the explanation of this and other similarities as a result of divergent (or convergent) evolution will remain a matter of personal taste unless a different approach to the evaluation of the significance of structural similarity is accepted” (Murzin, 1998). We are now at that crossroad, and it is up to us to replace the subjective nature of “personal taste” with the rigor of objective process.
